# Loss of BAP1 Expression Occurs Frequently in Intrahepatic Cholangiocarcinoma

**DOI:** 10.1097/MD.0000000000002491

**Published:** 2016-01-15

**Authors:** Juliana Andrici, Benjamin Goeppert, Loretta Sioson, Adele Clarkson, Marcus Renner, Albrecht Stenzinger, Michael Tayao, Nicole Watson, Mahtab Farzin, Christopher W. Toon, Ross C. Smith, Anubhav Mittal, Jaswinder S. Samra, Thomas J. Hugh, Angela Chou, Rita T. Lawlor, Wilko Weichert, Peter Schirmacher, Nicola Sperandio, Andrea Ruzzenente, Aldo Scarpa, Anthony J. Gill

**Affiliations:** From the Cancer Diagnosis and Pathology Research Group, Kolling Institute of Medical Research, St Leonards, NSW, Australia (JA, LS, AC, MT, NW, MF, CWT, AJG); Institute of Pathology, University Hospital Heidelberg, Heidelberg, Germany (BG, MR, AS, WW, PS); Department of Anatomical Pathology, Royal North Shore Hospital, St Leonards (LS, AC, MF, AJG); Histopath Pathology, North Ryde (CWT); Sydney Medical School, University of Sydney, Sydney (JA,CWT, RCS, AM, JSS, TJH, AJG); Upper Gastrointestinal Surgical Unit, Royal North Shore Hospital, North Shore Private Hospital, St Leonards; Discipline of Surgery, University of Sydney (AM, JSS, TJH); Macquarie University Hospital, Macquarie University, Sydney (JSS); Department of Anatomical Pathology, SYDPATH, St Vincents Hospitals, Darlinghurst, NSW, Australia (AC); Department of Pathology and Diagnostics, ARC-NET Research Center (RTL, NS, AS); Department of Surgery, University and Hospital Trust of Verona, Verona, Italy (AR); and Sydney Vital Translational Research Centre, Royal North Shore Hospital, Pacific Highway, St Leonards, NSW, Australia (AJG).

## Abstract

BRCA1-associated protein 1 (BAP1) is a deubiquitinating enzyme that functions as a tumor suppressor gene. Double hit *BAP1* inactivation has been reported in a range of tumor types, including intrahepatic cholangiocarcinoma (ICC), sometimes in association with germline mutation.

We performed immunohistochemistry for BAP1 on a well-characterized cohort of 211 ICC patients undergoing surgical resection with curative intent at 3 institutions based in 3 different countries. The median age at diagnosis was 65 years (range, 36.5–86) and 108 (51%) were men. Negative staining for BAP1 (defined as completely absent nuclear staining in the presence of positive internal controls in nonneoplastic cells) occurred in 55 ICCs (26%). BAP1 loss predicted a strong trend toward improved median survival of 40.80 months (95% CI, 28.14–53.46) versus 24.87 months (95% CI, 18.73–31.01), *P* = 0.059). In a multivariate model including age, sex, BAP1 status, tumor stage, tumor grade, lymphovascular invasion, and tumor size, female sex was associated with improved survival (hazard ratio [HR] 0.54; 95% CI, 0.34–0.85), while advanced tumor stage and lymphovascular invasion (HR 1.89; 95% CI, 1.09–3.28) correlated with decreased survival. In a multivariate analysis, high grade tumors were associated with BAP1 loss (odds ratio [OR] 3.32; 95% CI, 1.29–8.55), while lymphatic invasion was inversely associated with BAP1 loss (OR 0.36; 95% CI, 0.13–0.99).

In conclusion, we observed a trend toward improved prognosis in ICC associated with absent expression of BAP1 and an association of BAP1 loss with higher histological grade and absent lymphatic invasion. Female sex was associated with improved survival while advanced tumor stage and lymphatic invasion were associated with decreased survival.

## INTRODUCTION

BRCA1-associated protein 1 (BAP1) is a nuclear deubiquitinating enzyme in the ubiquitin C-terminal hydrolase family.^1,2^ It is encoded by the *BAP1* gene at 3p21.1 and together with other genes such as *ARID1A* and *PBRM1*, is involved in chromatin remodeling.^1,3^*BAP1* behaves as a true tumor suppressor gene and appears to follow a classic Knudson two-hit model.^1^ That is, germline *BAP1* mutation is associated with a newly recognized autosomal-dominant hereditary cancer syndrome, (OMIM #6143), characterized by uveal melanoma, mesothelioma, cutaneous melanocytic lesions, renal cell carcinoma, basal cell carcinoma, and intrahepatic cholangiocarcinoma (ICC);^4–7^ while biallelic inactivations including somatic mutations or deletions have also been reported in a range of tumors including uveal melanoma, mesothelioma, cutaneous melanocytic neoplasms, and clear cell renal carcinoma.^6–14^

Cholangiocarcinoma is a relatively rare malignancy of biliary epithelium, with a reported annual incidence of 8 per million in the United States.^15^ It accounts for approximately 3% of gastrointestinal cancers and is the second most common primary hepatobiliary malignancy after hepatocellular carcinoma.^16,17^ Cholangiocarcinomas may be classified as either intrahepatic or extrahepatic.^18^ Risk factors for cholangiocarcinoma include primary sclerosing cholangitis, liver fluke infestation, bile duct anomalies, biliary papillomatosis, chemical carcinogens including thorotrast and nitrosamines, obesity, nonalcoholic liver disease, and viral hepatitis.^18,19^ However, the majority of cholangiocarcinomas, at least in Western countries where liver fluke infection is rare, arise in the absence of predisposing factors.^18–20^ The outlook for cholangiocarcinoma is generally poor, with an overall 5-year survival of less than 5%.^15^

It has recently been suggested that germline *BAP1* mutations predispose to intrahepatic cholangiocarcinoma^5^ and somatic biallelic inactivating *BAP1* mutations have been reported in up to 25% of intrahepatic cholangiocarcinomas (ICC).^3,5,21^ Immunohistochemistry (IHC) for BAP1 appears to be a reliable marker of double hit inactivation of BAP1 in mesothelioma and melanoma ^1,22–25^ and phenotype–genotype correlations are rapidly emerging in both melanoma and mesothelioma. For example, in uveal melanoma loss of BAP1 expression is a strong predictor of adverse outcome,^23,24^ whereas in mesothelioma BAP1 loss is associated with a distinct phenotype of female sex, younger age onset, epithelioid morphology, and improved prognosis.^1,22,25^ Additionally, BAP1 loss in cells obtained by effusion cytology has been proposed as an adjunct to support a diagnosis of mesothelioma in select patients.^26^ However, to date, there has been no systematic study of BAP1 expression in ICC.

We therefore sought to investigate the clinical and pathological features associated with loss of expression of BAP1 as determined by IHC in a large multiinstitutional cohort of ICC specifically to explore if there are phenotype–genotype correlations for BAP1 loss in cholangiocarcinoma.

## METHODS

The computerized databases of the departments of anatomical pathology Royal North Shore Hospital, Sydney, Australia; University Hospital Heidelberg, Heidelberg, Germany; and University and Hospital Trust of Verona, Verona, Italy were searched for cases of definite intrahepatic cholangiocarcinoma diagnosed between January 1990 and August 2014. Cases where metastasis to the liver or primary pancreaticobiliary or extrahepatic origin were considered possible were excluded, as were cases in which no diagnostic material remained in formalin fixed paraffin embedded blocks. All cases were independently reviewed by experienced gastrointestinal pathologists to confirm the diagnosis of ICC and to restage according to the 7th edition 2009 AJCC staging system.^27^

A tissue microarray (TMA) containing duplicate 1-mm cores of formalin fixed paraffin embedded tumor tissue was constructed for the cases from Royal North Shore Hospital and Verona. For the cases from Heidelberg, whole sections were used. Immunohistochemistry (IHC) for BAP1 was performed on the TMA sections and whole slides using a mouse monoclonal anti-BAP1 antibody (clone C-4, cat no sc-28383, Santa Cruz Biotechnology, Dallas, Texas USA, dilution 1:100). The slides were stained on the Leica Microsystems Bond III autostainer (Leica Microsystems, Mount Waverley, Victoria, Australia) after heat-induced antigen retrieval for 30 minutes at 97°C in the manufacturer's alkaline retrieval solution ER2 (VBS part no: AR9640). A biotin-free polymer-based detection system (Define, VBS part no: DS 9713) was employed.

**FIGURE 1:**
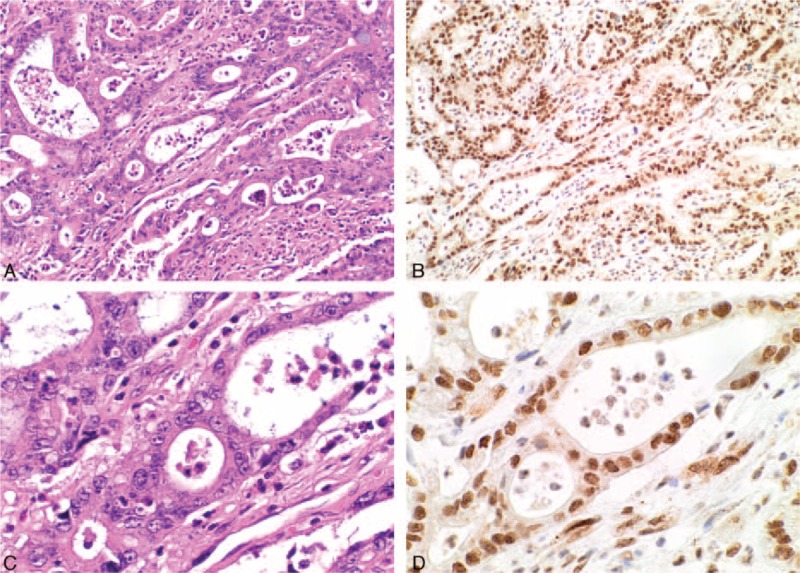
Serial H&E (A, C) and BAP1 IHC (B, D) stained sections from an intrahepatic cholangiocarcinoma demonstrating diffuse strong nuclear staining in both the neoplastic and nonneoplastic cells (original magnifications A, B ×100, C, D ×400).

**FIGURE 2:**
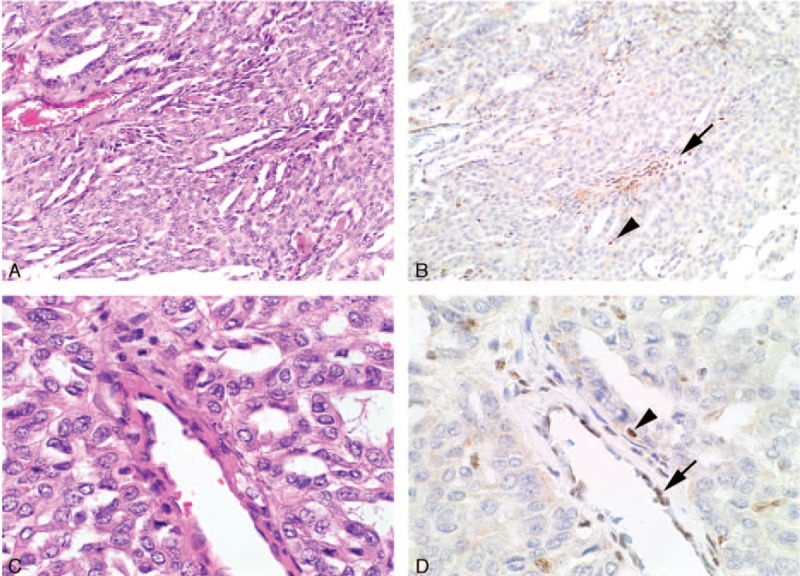
Serial H&E (A, C) and BAP1 IHC (B, D) stained sections from an intrahepatic cholangiocarcinoma that shows completely negative IHC staining for BAP1. In this case, the nonneoplastic endothelial cells (arrows) and lymphocytes (arrowheads) demonstrated preserved positive staining and serve as an internal positive control (original magnifications A, B ×100, C, D ×400).

**FIGURE 3:**
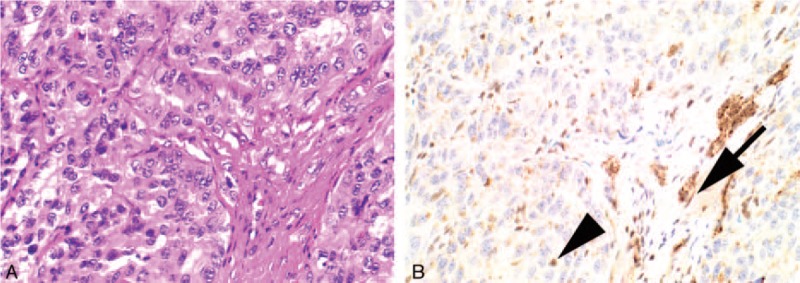
Serial H&E (A) and BAP1 IHC (B) stained sections from an intrahepatic cholangiocarcinoma. The neoplastic cells demonstrate completely negative nuclear staining for BAP1 with preserved positive staining for endothelial cells (arrows) and lymphocytes (arrowheads) which serve as an internal positive control. In this case, there is nonspecific cytoplasmic staining in both the neoplastic and nonneoplastic cells. For the purposes of interpreting BAP1 IHC, this cytoplasmic staining is ignored (original magnification A, B ×400).

BAP1 staining was interpreted by a single observer (AG) who was blinded to all clinical and other data at the time of assessment. Negative staining was defined as completely absent nuclear staining in all neoplastic cells in the presence of a positive internal control (nonneoplastic cells such as lymphocytes or stromal cells), as illustrated in Figure 1. Positive staining was defined as positive nuclear staining in any neoplastic cells (arbitrarily defined as greater than 2% of any definitely neoplastic cells) as illustrated in Figure 2. If the staining could not be interpreted on the TMA sections, due to insufficient material or the absence of an internal positive control, the result was considered indeterminate and repeated on whole mount sections (n = 5 cases). Cytoplasmic staining was considered nonspecific and disregarded (Figure 3). That is cases which demonstrated cytoplasmic staining only with negative nuclear staining were considered negative.

Overall survival time was obtained from medical records and publicly available death notices. Survival was calculated by Kaplan–Meier analysis and compared using the log-rank test. Characteristics of patients from the 3 cohorts, as well as those of BAP1 negative and BAP1 positive patients, were compared using Fischer exact test or Pearson *χ*^2^ to calculate *P* values for categorical variables, or the Mann–Whitney test for age, survival, and tumor size. Univariate regression analysis examined the impact of each variable individually on overall survival. Multivariate Cox regression proportional hazards analysis was then used to explore the effect of BAP1 status on overall survival, adjusted for age at diagnosis, sex, stage, lymphatic invasion, vascular invasion, tumor grade, and tumor size. Statistical analyses were performed using IBM SPSS Statistics v22. Statistical significance was defined as *P* < 0.05. This study was approved by the Northern Sydney Local Health District medical ethics review board.

## RESULTS

The study group included 211 patients with confirmed intrahepatic cholangiocarcinoma who underwent surgery with curative intent, comprising 49 patients from the Australian cohort, and 81 patients each from the Italian and German cohorts. The clinical and pathological details are summarized in Table 1. Briefly, the mean age at diagnosis was 65 years (range, 36.5–86 years) and 108 (51%) were men. The median survival was 29.37 months (95% confidence interval [CI], 21.61–37.13 months). Loss of BAP1 expression was present in 55 (26%) patients. There were some differences between the 3 cohorts from the different centres. The mean age of the German cohort was younger (62 years) than the Australian (76 years) or Italian (66 years) cohorts, *P* < 0.001. Patients in the German cohort also had larger tumors (mean, 70 mm) than patients in the Australian (50 mm) and Italian (45 mm) cohorts, *P* = 0.001. Lymphatic invasion was more prevalent in the Italian (37%) and German (40%) cohorts, compared with the Australian cohort (18%).

**TABLE 1:**
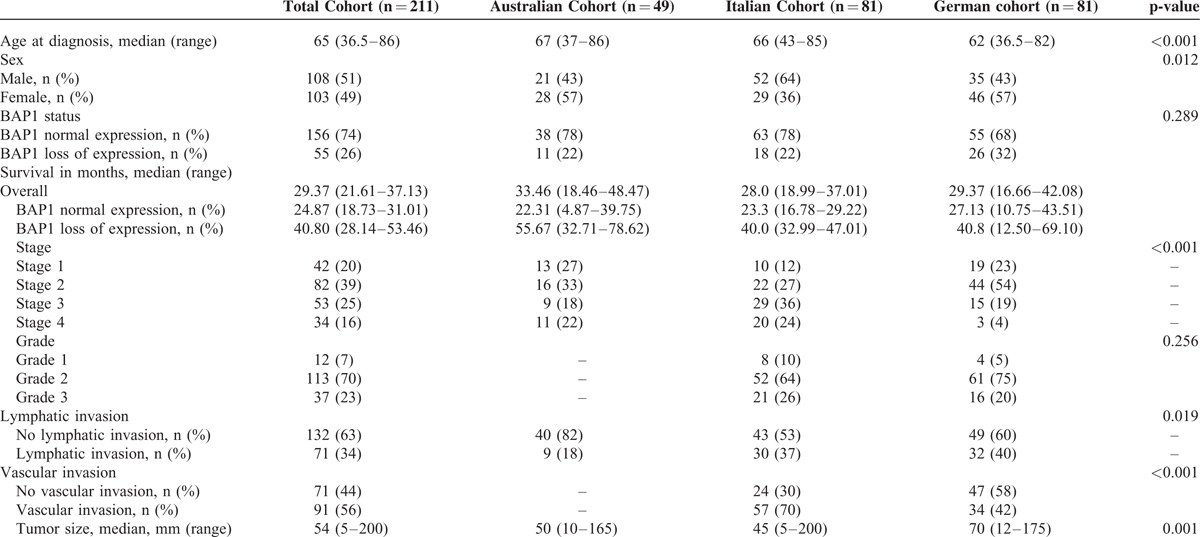
Patient Characteristics of Study Cohort

**FIGURE 4:**
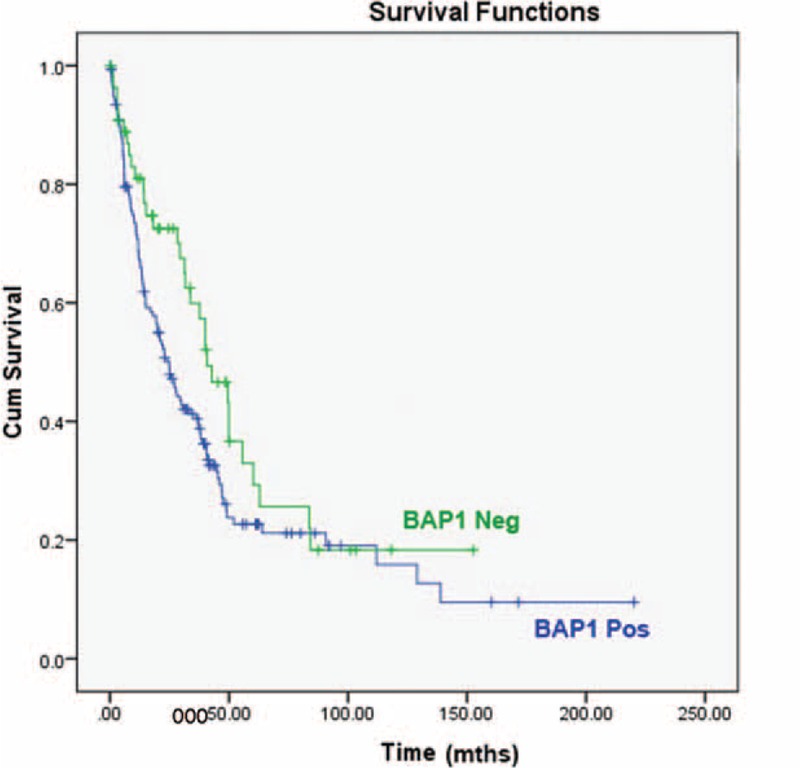
Kaplan–Meier survival curves demonstrate a trend toward improved survival in BAP1 IHC negative cholangiocarcinoma (*P* = 0.059).

The Kaplan–Meier survival curves by BAP1 status for the entire cohort are presented in Figure 4. The median overall survival for the cohort was 29.37 months (95% CI, 21.61–37.13 months). BAP1 IHC positive ICCs demonstrated worse median survival, 24.87 months (95% CI, 18.73–31.01 months), compared with BAP1 IHC negative cases, 40.80 months (95% CI, 28.14–53.46 months), but this did not reach statistical significance (*P* = 0.059). Multivariate analysis in a model which included age, sex, BAP1 status, tumor stage, tumor grade, lymphatic invasion, vascular invasion, and tumor size (Table 2) found only a slight trend toward increased survival associated with loss of BAP1 expression, which was not statistically significant (HR 0.89; 95% CI, 0.55–1.45; *P* = 0.645). The multivariable model only included the Italian and German cohorts (n = 162), as the Australian cohort was missing data on grade and vascular invasion. Female sex predicted better survival outcomes (HR 0.54; 95% CI, 0.34–0.85; *P* = 0.007), whereas Stage 2 tumors (HR 2.19; 95% CI, 1.12–4.27; *P* = 0.022), Stage 3 tumors (HR 2.68; 95% CI, 1.27–5.65; *P* = 0.010), Stage 4 tumors (HR 2.95; 95% CI, 1.11–7.89; *P* = 0.031), and lymphatic invasion (HR 1.89; 95% CI, 1.09–3.28; *P* = 0.023) correlated with decreased patient survival (Table 3). High grade tumors (HR 1.68; 95% CI, 1.10–2.57; *P* = 0.017) and vascular invasion (HR 1.49; 95% CI, 1.02–2.19; *P* = 0.039) were associated with decreased survival on univariate regression, but not in the multivariate analysis.

**TABLE 2:**
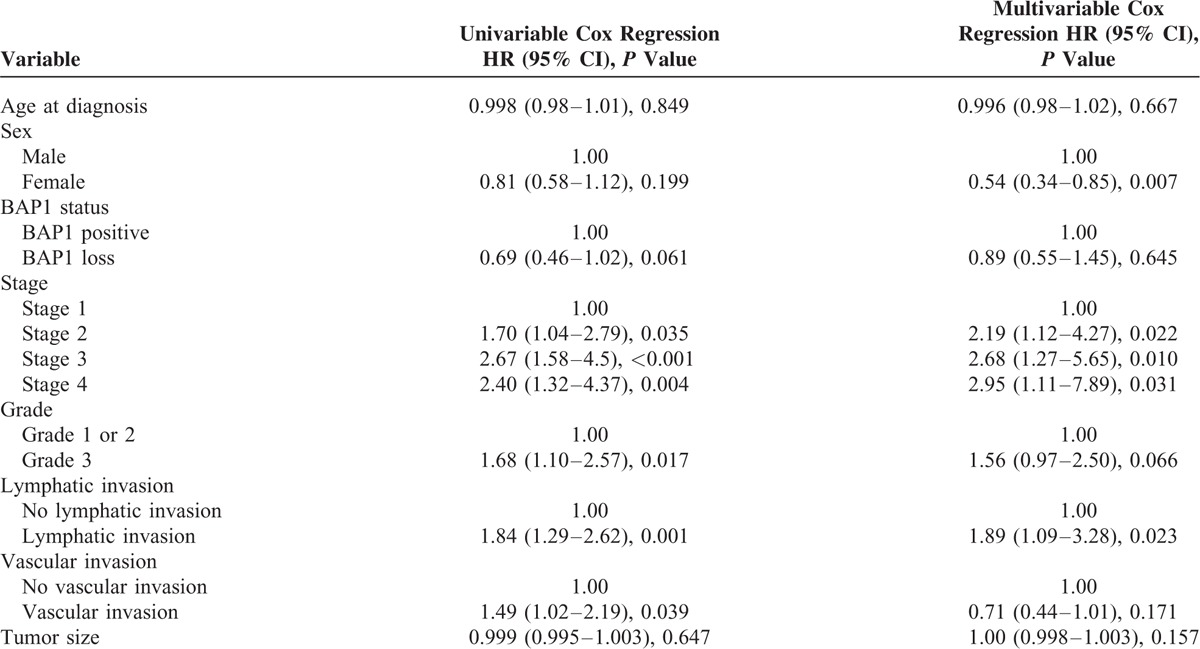
Univariate (n = 211) and Multivariate (n = 162) Analysis for Cholangiocarcinoma Patients for Overall Survival

**TABLE 3:**
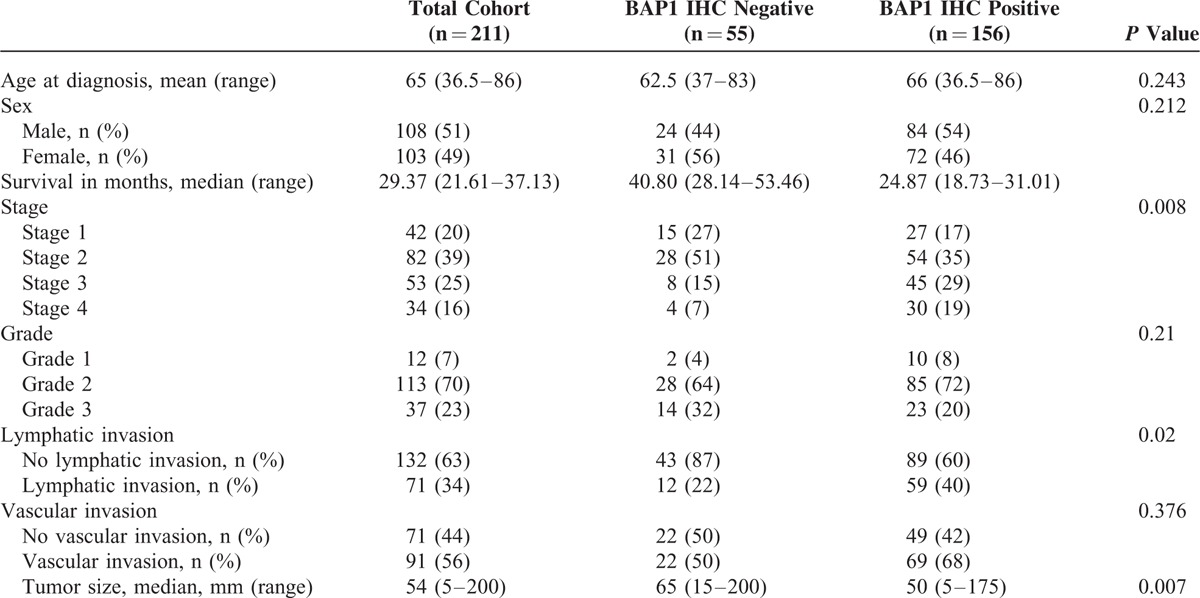
Patient Characteristics by IHC Staining

Tables 3 and 4 summarize patient characteristics by BAP1 status. On univariate analysis, patients with BAP1 loss were less likely to present with more advanced tumors—Stage 3 tumors (odds ratio [OR] 0.32; 95% CI, 0.12–0.85; *P* = 0.023), Stage 4 tumors (OR 0.24; 95% CI, 0.07–0.81; *P* = 0.022)—or with lymphatic invasion (OR 0.42; 95% CI, 0.21–0.86; *P* = 0.018). A small but statistically significant association between BAP1 loss and tumor size (OR 1.01; 95% CI, 1.003–1.02; *P* = 0.0008) was also observed on univariate analysis. On multivariate analysis, however, only the inverse relationship with lymphatic invasion remained statistically significant (OR 0.36; 95% CI, 0.13–0.99; *P* = 0.049). The multivariate analysis also found higher histological grade to be positively associated with BAP1 loss (OR 3.32; 95% CI, 1.29–8.55; *P* = 0.013).

**TABLE 4:**
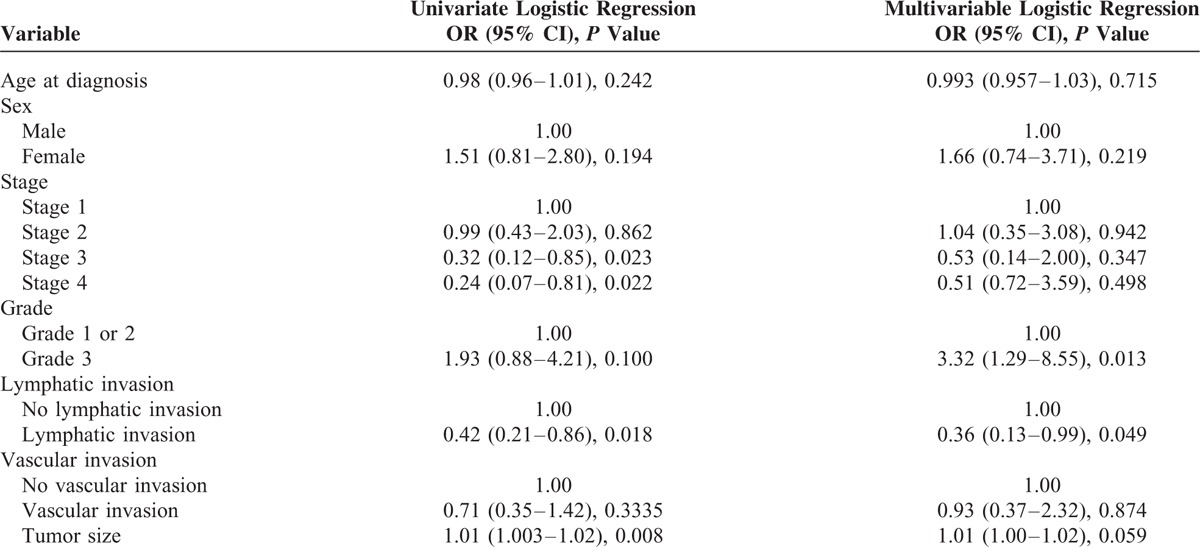
Univariate and Multivariate Analysis for Associations Between Clinicopathological Characteristics and BAP1 loss

## DISCUSISON

Here we report, for the first time, the incidence of negative staining for BAP1 in intrahepatic cholangiocarcinoma and the results of a multivariate analysis on the impact of BAP1 loss on overall survival. Our study included 211 cholangiocarcinoma cases from 3 major institutions in 3 different countries and diagnosed by experienced GIT pathologists. We demonstrate that complete loss of staining for BAP1 occurs in 26% of intrahepatic cholangiocarcinomas. Although our study was not intended or designed to assess the sensitivity and specificity of BAP1 IHC for *BAP1* mutation, our incidence of negative staining for BAP1 is very similar to the rate of inactivating mutations reported by Jiao et al^3^ (25%) and Chan-On et al^21^ (22.2%), suggesting that loss of IHC staining for BAP1 is likely to correlate strongly with *BAP1* mutation in ICC as it has been proven to do in mesothelioma and uveal melanoma.^1,22,24–27^

On univariate analysis, patients with BAP1 loss were less likely to present with more advanced tumors—Stage 3 tumors (OR 0.32; 95% CI, 0.12–0.85; *P* = 0.023), Stage 4 tumors (OR 0.24; 95% CI, 0.07–0.81; *P* = 0.022) or with lymphatic invasion (OR 0.42; 95% CI, 0.21–0.86; *P* = 0.018), but were more likely to present with larger tumors (OR 1.01; 95% CI, 1.003–1.02; *P* = 0.0008). On multivariate analysis, only association with lymphatic invasion remained statistically significant, while an association between higher histological grade and BAP1 loss gained statistical significance (OR 3.32; 95% CI, 1.29–8.55; *P* = 0.013). It is also interesting to note that BAP1 loss demonstrated some association (albeit not statistically significant) with female sex (OR 1.66; 95% CI, 0.74–3.71; *P* = 0.219) and younger age (OR 0.99; 95% CI, 0.96–1.03; *P* = 0.715). There was also a strong trend toward prolonged survival in BAP1 IHC negative cases (median survival of 40.80 months (95% CI 28.14–53.46 months) versus 24.87 months (95% CI 18.73–31.01 months) for BAP1 IHC positive cases, but this just failed to reach statistical significance (*P* = 0.059). This is in contrast to melanoma where BAP1 loss is significantly associated with worse prognosis,^23,24^ but similar to mesothelioma where BAP1 loss is associated with improved overall prognosis.^25^ Presumably this indicates that pathways other than those associated with BAP1 mutation are more lethal in mesothelioma but less lethal that BAP1 associated pathways in uveal melanoma.

It is also interesting to note that BAP1 loss is associated on multivariate analysis with tumors of higher histological grade and larger tumor size yet trended toward improved survival. We and others have previously demonstrated that in mesothelioma, BAP1 loss is associated with the epithelioid histologic subtype,^25,26,28^ and it is possible that in cholangiocarcinoma there may exist an association between BAP1 status and a morphology which includes solid more solid tumors which would be interpreted as higher grade. However on review of our BAP1 IHC negatives cases, we were unable to identify any distinctive morphological features. Our finding of some association between BAP1 loss and improved survival is in contrast with Jiao et al,^3^ who reported better 3-year survival rates among *BAP1* wild-type cholangiocarcinoma patients. However, we note that their findings did not reach statistical significance, they did not perform BAP1 IHC and that their study cohort was significantly smaller than ours (n = 32 vs n = 211).

The frequent finding of loss of BAP1 expression in ICC has several implications. First, it may be diagnostically important for surgical pathologists. It can be difficult to distinguish metastatic adenocarcinoma to the liver from primary intrahepatic cholangiocarcinoma as there are no specific IHC markers for primary ICC. Loss of IHC staining for BAP1 which occurred in 26% of ICC in our study could potentially be used to support a diagnosis of ICC when a patient presents with a liver lesion and the differential diagnosis is between metastatic carcinoma and ICC—particularly if mesothelioma (which at this stage appears to be the only other tumor with glandular or gland-like differentiation which shows a significant incidence of BAP1 loss)^25,26,28^ can be excluded by other means.

It has been suggested that ICC may be a component of the hereditary cancer syndrome associated with germline BAP1 mutations.^5^ Therefore, it is possible that IHC for BAP1 may play a role in triaging formal genetic testing for germline BAP1 mutation in patients presenting with cholangiocarcinoma. That is, if a tumor shows positive staining for BAP1 then germline *BAP1* mutation can be considered unlikely, whereas if a tumor shows negative staining for BAP1 then *BAP1* mutation is not excluded and formal counselling and genetic testing may be warranted in patients considered at high risk for hereditary disease for example due to onset at a young age or a family history of *BAP1*-associated malignancy such as mesothelioma, uveal melanoma, or cholangiocarcinoma. The incidence of germline *BAP1* mutations in cholangiocarcinoma is not currently known; however, the incidence of germline *BAP1* mutations in malignancies such as mesothelioma and metastasising uveal melanoma has been estimated at 1 to 2% or less.^22,27^ It is likely that the rate of germline *BAP1* mutations to be lower in cholangiocarcinoma than that found in mesothelioma and uveal melanoma, given that somatic mutations resulting in BAP1 loss occur in approximately half of mesotheliomas^1,22,28,29^ and in up to 84% of uveal melanomas,^30^ which is higher than the rate of BAP1 loss so far reported in cholangiocarcinoma.^3,21,31^ Given that real rate of germline *BAP1* mutations is likely to be less than 1%, it is reasonable that formal genetic testing for *BAP1* mutation be reserved for those patients who are considered high-risk based on family and personal history and demonstrate loss of BAP1 expression by IHC.

We observed a statistically significant association between female sex, lymphatic invasion, and tumor stage and survival. However, despite our large cohort our study might not be sufficiently powered to observe other potential relationships, especially when analyzing subgroups. The subgroup of BAP1-negative patients, for example, only consisted of 55 patients. We combined in our analyses cholangiocarcinoma patients from 3 separate institutions. While the BAP1 staining was interpreted by a single experienced pathologist (AG) who was blinded to all clinical data, the 3 cohorts were from different institutions and had slightly different patient characteristics. While the study patients all underwent surgery with curative intent, there may be differences in selection criteria for curative surgery and the use of adjuvant therapy at the different institutions. The fact that the study only included patients who were candidates for surgical resection with curative intent, as well as the potential for different selection criteria for treatment between institutions and surgeons, raises the possibility of selection bias. Other limitations of this study include its retrospective nature and the fact that IHC was performed on TMA rather than whole sections for many cases and therefore may have missed heterogeneous staining.

In conclusion, we report an incidence of negative IHC staining for BAP1 in cholangiocarcinoma of 26% which is keeping with the reported incidence of *BAP1* double hit inactivation as detected by molecular means of 22.2 to 25%.^3,21^ We suggest that there is a strong trend toward improved survival for BAP1 IHC negative cholangiocarcinomas but this trend just failed to reach statistical significance (*P* = 0.059) even in our large cohort of 211 resected cholangiocarcinomas. Other than for some association with higher histological grade, larger size, and absence of vascular space invasion, we could not identify a strong clinical or morphological phenotype for BAP1 IHC negative tumors undergoing surgery with curative intent.
